# The Role of Psychological Factors in Judo: A Systematic Review

**DOI:** 10.3390/ijerph19042093

**Published:** 2022-02-13

**Authors:** Carlo Rossi, Roberto Roklicer, Tatjana Tubic, Antonino Bianco, Ambra Gentile, Marko Manojlovic, Nemanja Maksimovic, Tatjana Trivic, Patrik Drid

**Affiliations:** 1 Sport and Exercise Sciences Research Unit, University of Palermo, 90144 Palermo, Italy; rcarlo97@hotmail.com (C.R.); antonino.bianco@unipa.it (A.B.); ambra.gentile91@gmail.com (A.G.); 2 Faculty of Sport and Physical Education, University of Novi Sad, 21000 Novi Sad, Serbia; roklicer.r@gmail.com (R.R.); tttubic@gmail.com (T.T.); markomanojlovic1995@gmail.com (M.M.); nemanjamaksimovic1998@gmail.com (N.M.); ttrivic@yahoo.com (T.T.)

**Keywords:** judo, motivation, mental toughness, anxiety, psychological preparation, mood state, performance

## Abstract

(1) Background: Psychological parameters are relevant in the practice of judo. Previous studies have shown that parameters such as anxiety or motivation can have a negative or positive impact on the athlete’s performance and general well-being, depending on the athlete’s perception. This systematic review aimed to summarize the studies examining the influence of various psychological parameters on well-being and performance in judo athletes; (2) Methods: We followed preferred reporting elements for systematic reviews and meta-analyses. We searched the Web of Science database for studies that explained the role of these parameters in elite athletes. Of the 286 articles initially identified, 17 met our eligibility criteria and were included in the review. In total, we analyzed data from 721 judo athletes; (3) Results: The studies found have demonstrated the impact of various psychological parameters during high-level performance and how these parameters can influence and lead an athlete to win or lose a competition. The feelings of tension, anger, anxiety, and nervousness were significantly increased in athletes who were facing defeat, while a decrease in the same segments and an increase in motivation among athletes who were experiencing better performance was observed. Further research under standardized conditions is needed to better understand the effects of these parameters on judo athletes; (4) Conclusions: Considering the athlete’s psychological state can affect performance, and it is therefore important to monitor and train these factors.

## 1. Introduction

Judo is an intermittent combat sport, dependent on anaerobic and aerobic metabolism and characterized by fast muscular actions [1]. The fight has a maximum duration of 5 min and the winner is judoka, who obtains the highest score or throws the opponent on his back (Ippon). It is a sport of unpredictability and is classified into weight categories [2]. Judo athletes commonly use rapid weight-reduction methods in the days prior to competition, in order to compete in a lower category [2]. Higher self-esteem indicates positive self-assessment, while low self-esteem refers to unfavorable self-opinion [3], and this factor plays a very important role in sport.

Anxiety is a state of discomfort often accompanied by somatic signs and symptoms of tension, and focused on possible failures, misfortunes, or dangers [4]. State anxiety refers to relatively unpleasant sensations of tension accompanied by activation of the autonomic nervous system [5]. On the other hand, pre-competition anxiety is the feeling of anxiety symptoms and is largely prevalent among athletes of all levels participating in many sports [6]. Martens et al. [7] proposed the multidimensional theory of anxiety to explain pre-competitive sports anxiety. The theory states that anxiety is composed of two parts, somatic and cognitive anxiety, which might affect performance considerably.

It is possible to separate a cognitive side of anxiety, named “cognitive anxiety”, caused by negative expectations of success or a negative self-assessment [8]. Conversely, the physical side of anxiety is somatic anxiety, which refers to the physiological and affective components of anxiety developed by autonomic arousal [9]. There are studies [9,10] suggesting that rapid weight loss can lead to increased motivation, decreased self-efficacy, and worsening mood. Mood refers to the set of positive and negative feelings that vary in intensity and duration [11], such as depression, tension, anger, fatigue, confusion, and vigor. Studies have shown a positive relationship between mood state and the “iceberg” profile (increased stamina and reduced other feelings) and sports performance [12,13,14]. According to Rouveix et al. [15], mood status can be changed due to alterations in body composition. This type of investigation might help judo coaches to increase knowledge about psychological adaptations that an elite judoka may experience during their competitive career.

Several scales estimating psychological parameters in judo athletes have been used, such as the Brunel Mood Scale (BRUMS) for the assessment of mood, the State-Trait Anxiety Inventory (STAI) questionnaire -T, Food Craving Questionnaire-Trait (FCQ-T), Restraint Scale (RS), Eating Attitude Test (EAT- 40), and the Profile of Mood States questionnaire (POMS).

Therefore, in this systematic review, we have evaluated various psychological parameters and to what degree they can influence performance in judo.

## 2. Materials and Methods

### 2.1. Literature Search Strategy

**Figure 1:**
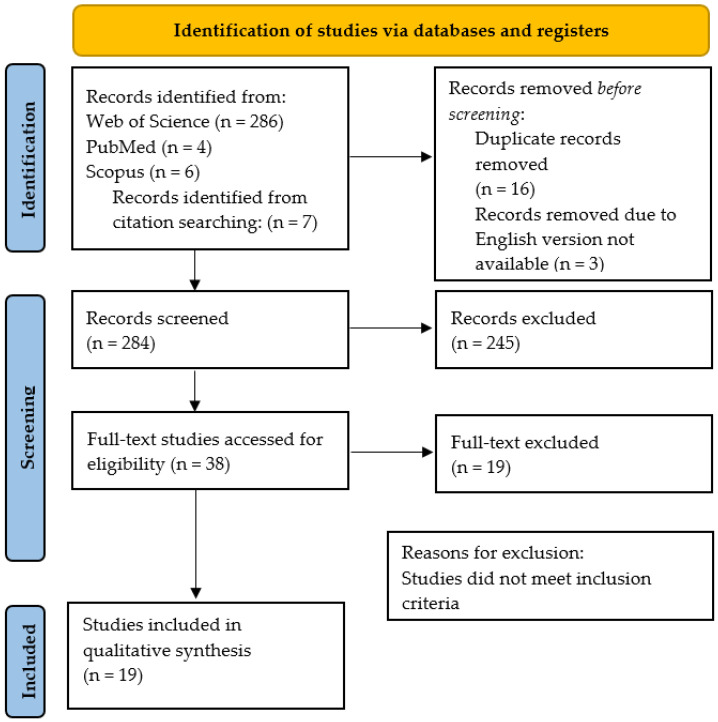
Flowchart PRISMA.

To ensure a transparent and comprehensive report, the guidelines for systematic reviews and meta-analysis were followed (PRISMA). To conduct this review, the Web of Science, PubMed, and Scopus databases were explored for the collection of articles. Additionally, articles from other sources were included as long as they were relevant for our study. The following string was applied: “Judo” AND “psychology”, “Judo” AND “psychological preparation”, “Judo” AND “anxiety”, “Judo” AND “mental toughness”, “Judo” AND “motivation”. The screening of the articles was carried out following three phases: reading the title, reading the abstract, and reading the full text. Between the two investigators, a third party independently considered the ongoing process and discussed the decision with the other researchers. Screening processes were summarized within the flowchart PRISMA, as shown in Figure 1.

### 2.2. Inclusion and Exclusion Criteria

Only original articles written in English and published in peer-reviewed journals were considered for inclusion in this review. The cut-off date for the publication period was set from the year 2000 to 2021. Various publication formats such as reviews, meta-analyses, citations, scientific conference abstracts, opinion articles, books, book reviews, statements, letters, editorials, non-peer reviewed journal articles, and commentaries were excluded. We considered studies with elite and junior and senior athletes, and both genders were eligible to be included in the review. Eligible items had to be conducted in judo athletes and had to include the measurement of psychological parameters within them (e.g., with rating scales). Both qualitative and quantitative articles were considered.

### 2.3. Data Extraction

Critical information on the included studies was delineated through tables (Microsoft Word 2013, Microsoft, London, UK), while a narrative description was performed to analyze the included literature on the topic. Some studies in the table were presented in narrative form, while others used signs that were explained in a legend, which provided details about a particular study that extended beyond the tabular explanation narrated in the results section. The data retrieved from the included articles concerned the influence of psychological parameters in judo and how they can, in turn, affect sports performance.

### 2.4. Risk of Bias Assessment

Risk of bias was assessed through the Downs and Black [16] checklist for quantitative research and the JBI Appraisal Checklist for qualitative research [17], evaluating the quality of original research articles included in the current review. The Downs and Black checklist is made up of 27 ‘yes’-or- ‘no’ questions across five domains (Reporting, External Validity, Internal Validity—bias, Internal Validity—confounding, Power).

The JBI Appraisal Checklist for qualitative research consists of 10 items with four possible answers (Yes/No/Unclear/Not Applicable): congruity between philosophical perspective and the research methodology (Item 1), congruity between research methodology and the research question (Item 2), congruity between research methodology and the methods used (Item 3), congruity between research methodology and the representation and data analysis (Item 4), congruity between research methodology and the interpretation of the results (Item 5), cultural or theoretical location of the researcher (Item 6), influence of the researcher on the research (Item 7), representativity of the sample (Item 8), ethical approval (Item 9), flows in the conclusion (Item 10).

Two independent researchers (MM and AG) completed the Downs and Black checklist and the JBI for included articles. The studies evaluated through the Downs and Black checklist were then distinguished into groups and labeled as ‘high quality’. Concerning the studies evaluated through the Downs and Black checklist, out of 16 quantitative studies, 11 were judged as “poor quality studies” and five as “medium quality studies”. The three qualitative studies were included in the systematic review with a good level of recommendation (for more information about risk of bias results, see Tables S1 and S2).

## 3. Results

The characteristics of the studies included in the systematic review are visible in Table 1 and Table 2 (for more information on the competitive level and training experience of athletes, see Table S3). The results show that weight loss has a meaningful effect on all components of mood status. Five articles present the relationship between anxiety and various performance of judokas.

Only two studies emphasize the importance of motivation in judo athletes. Coach autonomy support has a major role in self-determined motivation of young judokas [33]. Silva et al. [32] assessed the profile of mental toughness in judo.

## 4. Discussion

The goal of this study was to examine the impact of different psychological parameters on sports performance in judo athletes. Anger, tension, confusion, depression, fatigue, vigor, anxiety, motivation, and mental toughness have been identified as crucial psychological aspects that discriminate successful and less successful judokas.

Weight reduction definitely leads to changes in mood state segments. In general, weight loss increases negative mood state factors (anger, confusion, tension, depression, and fatigue) and decreases vigor, which is a positive component. The use of weight loss techniques causes elevated anger, confusion, and depression in young wrestlers [37,38,39]. Also, in some combat sports, weight reduction negatively affects the level of fatigue, tension, and total mood disturbance before competition [40,41]. On the other hand, vigor was inversely associated with the application of rapid weight loss [42]. The literature presented that weight loss increases the total mood disturbance of senior and junior judo athletes [18]. Also, it is significant to mention that weight reduction probably has a stronger effect on mood state alterations in male than in female judokas [18,19]. Therefore, it is suggested that female participants have a better psychological response to weight loss compared to males. Authors describe this phenomenon as a psychological stress potentially induced by the actual weight loss in males, while in females, it might be provoked by anxiety caused by the general concept of weight loss before the actual start of the physical process [18]. Mood variability is an important determinant of the manifestation of sports potential. There are contradictory results on the influence of anger on athlete performance. Páez-Ardila et al. [34] showed that judokas with increased anger were those who were defeated, while higher levels of anger improved the chances of better performance in individual and team sports [42,43]. Furthermore, the vigor deficit reduces the probability of winning [44]. Elevated tension, which is also a consequence of weight loss in judokas, has a positive effect on the performance of athletes [43,44]. On the contrary, lower levels of depression are desirable before the judo championship [43]. The control of all aspects of mood state requires great attention in judo and other sports because they can significantly contribute to the final outcome in competitive matches.

Anxiety is one of the most important psychological variables in modern sport. Much research has been devoted to understanding the relationship between anxiety and sports performance. Judo athletes marked as losers had higher levels of cognitive anxiety, but winners had more self-confidence [25]. Similarly, combat athletes who won their matches reported less cognitive and somatic anxiety and more self-confidence than those who lost [45,46]. State and trait anxieties were lower for winners than losers [47,48,49]. Competition at a higher level caused an increase in somatic and cognitive anxiety and a decrease in self-confidence in judokas [24]. In elite basketball athletes, playing against a tougher opponent has also raised both aspects of anxiety [50]. Age and gender are relevant predictors of judoka anxiety. Age was inversely related to cognitive anxiety and junior females had higher anxiety scores than males [27,29]. Freire et al. [51] found that older jiu-jitsu athletes presented lower magnitudes of cognitive anxiety. In accordance with the results of this study, males were less anxious than females in sports such as wrestling, karate, and taekwondo [52,53]. It is obvious that anxiety has a huge impact on the performance of judo athletes. Mental training techniques are necessary to monitor and maintain optimal levels of anxiety. Additionally, overtraining leads to increased emotional stress and exhaustion in junior judokas [28]. Intensified training periods can elevate psychological stress and fatigue in professional football players [54].

Another meaningful psychological parameter is motivation. Judo athletes with a high level of motivation have superior mental efficiency and a lower impulsivity index than those who are less motivated [31]. Stronger motivation to exercise was negatively correlated with mental burnout in weightlifters [55]. As in the presented review article, coaches’ autonomy support has a significant effect on the self-determined motivation that positively affects the sports performance of young athletes [56,57,58]. Combativity is an attribute of mental toughness typical for judokas [32]. Combativeness includes a tendency to dominate the rival, an energetic and aggressive attitude towards fighting in order to neutralize the opponent’s game [32]. The importance of combativity in judo has been previously shown in the literature [59]. Successful judokas and other combat athletes are characterized by a high level of mental toughness [35,60,61]. As with anxiety, age and gender were important determinants of mental toughness [35]. Researchers emphasize that gender differences are present due to different perceptions of females and males in the process of socialization, while maturity and training experience explain the higher values of mental toughness in older athletes [35]. Moreover, mental toughness has been responsible for increasing perfectionistic strivings and decreasing perfectionistic concerns in judo and other sports [30,62]. Finally, the concept of a dual career can be a problem for female judo athletes [33]. Skrubbeltrang et al. [63] demonstrated that the overlap between school and sports in students with higher socioeconomic statuses probably leads them to focus more on learning. 

This review paper has several relevant limitations. There are a small number of articles that analyze the relationship among significant psychological parameters, such as motivation and mental toughness and the performance of judokas. Additionally, the search strategy for articles was limited to those written in English language. The sincerity of athletes in completing the psychological questionnaires could have influenced the final results of all research. There is an obvious lack of studies that focus on the impact of the mentioned psychological segments on aerobic, anaerobic, or technical performance in judo athletes. Future research should go in that direction.

## 5. Conclusions

In summary, the results of the study show that sport psychology has an important role in the manifestation of judoka performance. The components of mood state (anger, tension, confusion, depression, fatigue, and vigor), anxiety, motivation, and mental toughness have been identified as key psychological parameters potentially affecting the outcome of judo matches. Successful judokas likely possess higher vigor, good control of negative aspects of mood state, less anxiety, and increased values of motivation and mental toughness. Therefore, cooperation between coaches and sports psychologists is necessary to monitor the mentioned aspects due to their impact on judo performance.

## Figures and Tables

**Table 1:** Mood state alterations in judo athletes.

Authors	Age	Sample	Methods/Treatment	Questionnaire	Parameters	Outcomes	
Yoshioka et al. [18]	19.5 ± 0.6 19.0 ± 0.7	*n* = 43 M = 27 F = 16	Weight reduction Questionnaire	POMS	*Males*		
					Fatigue	↑** in WR group (F-U)	
					Tension	↑* in WR group (F-U)	
					Vigor	↓* in WR group (F-U)	
					TMD	↑* in WR group (F-U)	
					*Females*		
					All variables	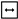	
Koral, Dosseville [19]	Mean age = 17	*n* = 20 M = 10 F = 10	DIET Questionnaire	POMS	Mood state	T1	T2
					Confusion	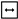	↑* (M, F)
					vigor	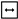	↓* (M, F)
					Tension	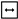	↑* (F)
Hernández et al. [20]	Mean age = 20.7	*n* = 10 M = 5 F = 5	Questionnaire Mood state Likert-type scale		BL	F-U	
				Fatigue	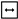	↓*	
				Tension	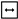	↑*	
				Vigor	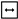	↑*	
Fortes et al. [21]	Mean age = 21.5	*n* = 42 M = 42	Weight loss (EG) Questionnaire	POMS	Mood state	EG	CG
					Tension	↑*	↑*
					Depression	↑*	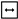
					Anger	↑*	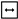
					Fatigue	↑*	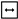
					Confusion	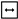	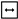
					Vigor	↓*	↑*
Chtourou et al. [22]	21 ± 1	*n* = 14 M = 14	RPE Scale and Hooper Questionnaire Shutter sprint Jump ability	POMS-f	Mood state	Morning	Afternoon
					Vigor	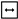	↑**
				Hooper-Q	Stress	↑**	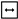
Isacco et al. [23]	24 ± 5	*n* = 20 (M, F)	Weight reduction: psychological profile during 5 successive fights (F1, F5) of a simulated judo competition Questionnaire	POMS	Mood state	Each of the dimensions (mood states) showed a significant time effect (*p* < 0.001) and significant group × time interactions at F4 and F5.	
					Tension		
					Depression		
					Anger		
					Vigor		
					Fatigue		
					Confusion		

Legend: F4—Fight number four; F5—Fight number five; WR—Weight reduction group; ↑**—Significant increase *p* < 0.01; ↑*—Significant increase *p* < 0.05; ↓*—Significant decrease *p* < 0.05; 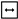
—Insignificant change; EG—Experimental group; CG—Control group; T1—Four weeks before the competition; T2—One day before the competition; POMS—Profile Of Mood States; POMS-F—Profile of Mood States French version; Hooper Q—Hooper questionnaire; F-U—Follow up measurement; BL—Baseline; *n*—Number of participants; M—Males; F—Females; TMD—Total mood disturbance.

**Table 2:** Influence of other psychological aspects on judokas.

Authors	Age	Sample	Questionnaire	Parameters	Outcomes			
Filaire et al. [24]	22.2 ± 1.6	*n* = 12 M = 1	STAI-Y-2 CSAI-2		Reg. Champ.		Interreg. Champ.	
				Y-2 Trait anxiety	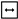		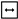	
				Y-1 State anxiety	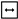		↑*	
				Cognitive A-state	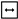		↑*	
				Somatic A-state	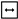		↑*	
				Self-confidence	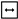		↓*	
Filaire et al. [25]	Age 22.2 ± 1.6	*n* = 18 M = 18	STAI-Y-2		Losers		Winners	
				Behavior type	B		A	
				Y-2 (trait anxiety)	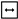		↑*	
				Behaviour pattern (Bortner)	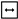		↑*	
				Y-1 (state anxiety)	↑*		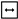	
				Somatic A-state/28	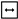		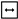	
				Cognitive A-state/28	↑*		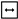	
				Self-confidence/36	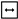		↑*	
				Solving problem factor/32 (Problem-focused strategies)	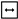		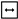	
				Self-blamed/16 (emotion-focused strategies)	↑*		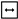	
				Avoidance/28 (emotion-focused strategies)	↑*		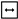	
				Social support approb/20 (emotion-focused strategies)	↑*		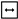	
				Positive re-evaluation/20 (emotion-focused strategies)	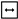		↑*	
Gillet et al. [26]	Mean age = 18.47	*n* = 101 M = 69 F = 32	EPSAS Adaptation of the Perceived Autonomy Support Scale for Exercise Settings to the sport setting.	Self-determined motivation before a competition	Significant correlation between intrinsic motivation and identified regulation (*p* < 0.001). The lowest correlation was obtained between intrinsic motivation and amotivation (*p* < 0.05). A significant indirect effect from coach autonomy support to situational motivation (*p* > 0.05) via contextual motivation (*p* < 0.01). Sobel test also showed that the indirect effect (via situational motivation) of contextual motivation on sport performance was statistically significant (*p* < 0.05).			
Kolayis et al. [27]	Age 20.53 ± 2.93	*n* = 126 M = 82 F = 44	CSAI-2 STAI	State anxiety Cognitive anxiety Somatic anxiety Self-confidence Self-esteem Education level	Significant positive correlation between the values of age and self-confidence (r: 0.256, *p* < 0.05), training age and self-confidence (r: 0.289, *p* < 0.05), state anxiety and cognitive anxiety (r: 0.435, *p* < 0.05), state anxiety and education level (r: 0.216, *p* < 0.05), state anxiety and somatic anxiety (r: 0.597, *p* < 0.05), cognitive anxiety and somatic anxiety (r: 0.578, *p* < 0.05), education level and competition ranking (r: 0.244, *p* < 0.05). A negative correlation was observed between the values of age and cognitive anxiety (r: −0.278, *p* < 0.05), age and education level (r: −0.376, *p* < 0.05), training age and state anxiety (r: −0.330, *p* < 0.05), training age and education level (r: −0.434, *p* < 0.05), training age and somatic anxiety (r: −0.280, *p* < 0.05), state anxiety and self-confidence (r: −0.652, *p* < 0.05), cognitive anxiety and self-confidence (r: −0.367, *p* < 0.05), education level and self-confidence (r: −0.220, *p* < 0.05), somatic anxiety and self-confidence (r: −0.470, *p* < 0.05).			
Noce et al. [28]	N.A. Junior (−18) Senior (+18)	*n* = 48 (M, F)	RESTQ-Sport scores		Pre-comp. Sen.	Pre-Comp Jun.	Post-comp Sen.	Post-comp Jun.
				General Stress	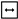	↑#	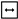	↑#
				Emotional Stress	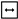	↑#	N.A.	N.A.
				Lack of Energy	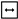	↑#	N.A.	N.A.
				Success	↑#	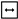	↑#	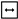
				General Well-being	↑#	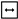	↑#	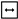
				Sleep Quality	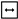	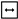	N.A.	N.A.
				Emotional Exhaustion	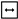	↑#	N.A.	N.A.
				Being in Shape	↑#	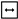	N.A.	N.A.
Molina et al. [29]	Junior (under −20) Senior (+20)	*n* = 98 M = 56 F = 42	STAI-T FCQ-T	STAI-T scores	The difference for STAI-T anxiety scores were significantly different between females and males only for juniors (*p* = 0.017).			
				FCQ-T subscales				
				Anticipation of positive reinforcement	In the anticipation of the positive reinforcement scale, juniors scored significantly higher than seniors (*p* = 0.001)			
				Anticipation of relief from negative states	In the anticipation of relief from the negative states scale, seniors scored higher than juniors (*p* = 0.01)			
Suárez-Cadenas et al. [30]	Aged between 16 and 69 Mean age = 28.73 ± 13.96	*n* = 118 F = 24 M = 94	SMTQ The Sport Multidimensional Perfectionism Scale	MT	Veterans scored higher than elite and sub-elite athletes on MT (*p* < 0.001).			
				Perfectionism	Perfectionistic strivings global scores only differed between veterans and sub-elite group (*p* < 0.001). Striving for perfection subscale showed that veterans scored higher than both elite and sub-elite athletes. Linear regression model showed that MT is positively associated with perfectionistic strivings (*p* < 0.001) and negatively associated with perfectionistic concerns (*p* < 0.001).			
Korobeynikov et al. [31]	N.A.	*n* = 25 M = 25	Level of motivation Questionnaire test	Mental state of high qualification judo athletes with different levels of motivation	Mental efficiency is significantly higher in the group of athletes with a predominance of motivation to achieve success (group 1) compared to a group of groups of judo athletes with average levels of motivation. Athletes with average levels of motivation to achieve success (group 2) and avoid a failure have significantly lower overall mental performance and discomfort compared to other groups. Athletes with high levels of motivation to achieve success revealed high stress resistance. The speed of response to stress factors is greatest in a group of athletes with a motivation to avoid failure (group 3) The impulsiveness index is significantly higher in the group of athletes with an average level of motivation			
Silva et al. [32]	Mean age = 24.6	*n* = 8 (M, F)	Interview protocol	Mental toughness in judo (elite and sub-elite athletes)	All subjects reported the importance of emotional regulation, resilience, self-confidence, attention regulation, self-motivation, and optimism. Nevertheless, combativity appears to be the only mental toughness attribute typical to judo.			
Kavoura, Ryba [33]	Mean age = 19.6	*n* = 6 F = 6	Interview	Identity tensions (dual career –plan for the future)	Some female judo athletes may experience identity tensions and lower their athletic aspirations in seeking to meet the new societal expectations embedded in the dual career discourse.			
Páez-Ardila et al. [34]	Age N.A.	*n* = 12 M = 12	Staxi Stai	Basal anger out Anxiety Anger	Significant statistical differences (*p* < 0.05) were found between winners and losers. Losers had higher levels of anger, while anxiety was higher for the winners. Statistically significant difference in basal anger out (*p* = 0.035) The subjects who were going to lose had a higher level of anger than those who won.			
Yasar, Turgut [35]	Mean age 20.65	*n* = 117 M = 63 F = 54	One-dimension mental toughness scale	Mental toughness	Respondents presented that the mental toughness is positively correlated with age (*p* = 0.007). The mean scores observed for mental toughness were higher in males compared to females (*p* = 0.032).			
Gordon et al. [36]	Age range = 20–28	*n* = 12 F = 7 M = 5	RWL Interview	Motivation to compete	Intrinsic motivation appears to be the most self-determined construction of motivation, which refers to performing an activity in order to obtain satisfaction and pleasure generated from participation.			
				Negative emotions and struggle	RWL negatively affected emotions. Struggling and anger were observed during the weight reduction procedures.			

↑*—Significant increase *p* < 0.05; ↓*—Significant decrease *p* < 0.05; c—Significantly different *p* < 0.001 compared to F1; Reg. Champ—Regional championship Interreg. Champ—Interregional championship; Pre-comp—Pre-competition; Post-comp—Post-competition; Jun—Junior; Sen—Senior; N.A.—Not available; ↑#—Significantly higher compared or juniors/seniors; 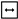
—Insignificant change; MT—Mental toughness; STAY-2—State trait anxiety inventory 2; CSAI-2—Competitive state anxiety inventory 2; RESTQ-Sport scores—Recovery-Stress Questionnaire; SMTQ—Sports Mental Toughness Questionnaire; STAI-T- State-Trait Anxiety Inventory; FCQ-T- Food Craving; STAI—State Trait Anxiety Inventory; STAI-Y-2—State-Trait Anxiety Inventory; EPSAS—Echelle des Perceptions du Soutien à l’Autonomie en Sport; Staxi—State trait anger expression inventory; Stai—State trait anxiety inventory; RWL –Rapid Weight Loss.

## Data Availability

Not applicable.

## Supplementary Materials

The following supporting information can be downloaded at: https://www.mdpi.com/article/10.3390/ijerph19042093/s1, Table S1: Risk of Bias of quantitative studies through the Downs and Black checklist; Table S2: Risk of Bias of quantitative studies through the JBI Appraisal Checklist; Table S3: Competitive level and training experience.

## References

[1] Franchini E., Del Vecchio F.B., Julio U.F., Matheus L., Candau R. (2015). Specificity of performance adaptations to a periodized judo training program. Rev. Andal. Med. Deporte.

[2] Artioli G.G., Franchini E., Nicastro H., Sterkowicz S., Solis M.Y., Lancha A.H. (2010). The need of a weight management control program in judo: A proposal based on the successful case of wrestling. J. Int. Soc. Sport Nutr..

[3] Baumeister R.F., Campbell J.D., Krueger J.I., Vohs K.D. (2003). Does high self-esteem cause better performance, interpersonal success, happiness, or healthier life-styles?. Psychol. Sci. Public Interest.

[4] Corman A.M. (2003). Dictionary of Psychology.

[5] Vingerhoets G. (1998). Perioperative anxiety and depression in open-heart surgery. Psychosomatics.

[6] Alexander D. (2009). Psychophysiological Effects of Pre-Competition Anxiety on Basketball Performance. Master’s Thesis.

[7] Martens R., Burton D., Vealey R.S., Bump L.A., Smith D.E., Martens R., Vealey R.S., Burton D. (1990). Development and validation of the Competitive State Anxiety Inventor y-2. Competitive Anxiety in Sport.

[8] Ingram R.E., Kendall P.C. (1987). The cognitive side of anxiety. Cognit. Ther. Res..

[9] Filaire E., Rouveix M., Pannafieux C., Ferrand C. (2007). Eating attitudes, perfectionism and body-esteem of elite male judoists and cyclists. J. Sports Sci. Med..

[10] Brito C.J., Roas A.F.C.M., Brito I.S.S., Marins J.C.B., Córdova C., Franchini E. (2012). Methods of body-mass reduction by combat sport athletes. Int. J. Sport Nutr. Exerc. Metab..

[11] Mcnair D.M., Lorr M., Droppleman L.F. (1971). Manual for the Profile of Mood States.

[12] Mujika I., Chaouachi A., Chamari K. (2010). Precompetition taper and nutritional strat- egies: Special reference to training during Ramadan intermittent fast. Br. J. Sports Med..

[13] Le Meur Y., Hausswirth C., Mujika I. (2012). Tapering for competition: A review. Sci. Sports.

[14] Fortes L.S., Oliveira S.F.M., Mendonçam L.C.V., Oliveira G.J.S., Paes P.P., Fonseca A.M.L.F.M.da. (2017). Does disordered eating decrease the agility and vertical jump in combat sports athletes?. Rev. Bras. Educ. Fis. Esporte.

[15] Rouveix M., Bouget M., Pannafieux C., Champely S., Filaire E. (2007). Eating attitudes, body esteem, perfectionism and anxiety of judo athletes and nonathletes. Int. J. Sports Med..

[16] Downs S.H., Black N. (1998). The feasibility of creating a checklist for the assessment of the methodological quality both of randomized and non-randomized studies of health care interventions. J. Epidemiol. Community Health.

[17] Lockwood C., Munn Z., Porritt K. (2015). Qualitative research synthesis: Methodological guidance for systematic reviewers utilizing meta-aggregation. JBI Evid. Implement..

[18] Yoshioka Y., Umeda T., Nakaji S., Kojima A., Tanabe M., Mochida N., Sugawara K. (2006). Gender differences in the psychological response to weight reduction in judoists. Int. J. Sport Nutr. Exerc. Metab..

[19] Koral J., Dosseville F. (2009). Combination of gradual and rapid weight loss: Effects on physical performance and psychological state of elite judo athletes. J. Sports Sci..

[20] Hernández R., Torres-Luque G., Olmedilla A. (2009). Relations among training volume; body weight; and profile of mood states for elite judoka during a competitive period. Percept. Mot. Ski..

[21] Fortes L.S., Lira H.A., Andrade J., Oliveira S.F., Paes P.P., Vianna J.M., Vieira L.F. (2018). Mood response after two weeks of rapid weight reduction in judokas. Arch. Budo.

[22] Chtourou H., Engel F.A., Fakhfakh H., Fakhfakh H., Hammouda O., Ammar A., Trabelsi K., Souissi N., Sperlich B. (2018). Diurnal variation of short-term repetitive maximal performance and psychological variables in elite judo athletes. Front. Physiol..

[23] Isacco L., Degoutte F., Ennequin G., Pereira B., Thivel D., Filaire E. (2020). Rapid weight loss influences the physical; psychological and biological responses during a simulated competition in national judo athletes. Eur. J. Sport Sci..

[24] Filaire E., Sagnol M., Ferrand C., Maso F., Lac G. (2001). Psychophysiological stress in judo athletes during competitions. J. Sports Med. Phys. Fit..

[25] Filaire E., Maso F., Sagnol M., Ferrand C., Lac G. (2001). Anxiety, hormonal responses, and coping during a judo competition. Aggress. Behav..

[26] Gillet N., Vallerand R.J., Amoura S., Baldes B. (2010). Influence of coaches’ autonomy support on athletes’ motivation and sport performance: A test of the hierarchical model of intrinsic and extrinsic motivation. Psychol. Sport Exerc..

[27] Kolayis H., Sari I. (2011). Anxiety; self-esteem and competition ranking of judokas. Arch. Budo.

[28] Noce F., Costa V.T., Szmuchrowski Soares D.S., de Mello M.T. (2014). Psychological indicators of overtraining in high level judo athletes in pre- and post-competition periods. Arch. Budo.

[29] Molina R.E., Rodríguez-Ruiz S., Gutiérrez-García C., Franchini E. (2015). Weight loss and psychological-related states in high-level judo athletes. Int. J. Sport Nutr. Exerc. Metab..

[30] Suarez-Cadenas E., Sretkovic T., Perales J.C., Petrovic J., Sterkowicz-Przybycien K., Batez M., Drid P. (2016). Mental toughness and perfectionism in judo: Differences by achievement and age. The relation between constructs. Arch. Budo.

[31] Korobeynikov G.V., Korobeynikova L.G., Romanyuk L.V., Dakal N.A., Danko G.V. (2017). Relationship of psychophysiological characteristics with different levels of motivation in judo athletes of high qualification. Pedagog. Phys. Cult. Sports.

[32] Silva V., Dias C., Corte-Real N., Fonseca A. (2018). Mental toughness attributes in judo: Perceptions of athletes. Cuad. Psicol. Deporte.

[33] Kavoura A., Ryba T.V. (2019). Identity tensions in dual career: The discursive construction of future selves by female Finnish judo athletes. Sport Soc..

[34] Páez-Ardila H.A., Lopes Campos I.S., Gouveia A. (2020). Evidence of the effect of winning or losing in levels of anger and anxiety in judo fighters. Av. Psicol. Latinoam..

[35] Yasar O.M., Turgut M. (2020). Mental toughness of elite judo athletes. Acta Med..

[36] Gordon Y., Souglis A., Andronikos G. (2021). Effect of weight restriction strategies in judokas. JPES.

[37] Marttinen R.H.J., Judelson D.A., Wiersma L.D., Coburn J.W. (2011). Effects of self-selected mass loss on performance and mood in collegiate wrestlers. J. Strength Cond. Res..

[38] Karninčić H., Baić M., Slačanac K. (2016). Mood aspects of rapid weight loss in adolescent wrestlers. Kinesiology.

[39] Slačanac K., Baić M., Karninčić H. (2021). The relationship between rapid weight loss indicators and selected psychological indicators on success of Croatian wrestlers. Arch. Budo.

[40] Brandt R., Bevilacqua G., Coimbra D., Pombo L.C., Miarka B., Lane A.M. (2018). Body weight and mood state modifications in Mixed Martial Arts: An Exploratory Pilot. J. Strength Cond. Res..

[41] Hall C.J., Lane M. (2001). Effects of rapid weight loss on mood and performance among amateur boxers. Br. J. Sports Med..

[42] Castor-Praga C., Lopez-Walle J.M., Sanchez-Lopez J. (2021). Multilevel evaluation of rapid weight loss in wrestling and taekwondo. Front. Sociol..

[43] Brandt R., Bevilacqua G.G., Crocetta T.B., Monteiro C.B., Guarnieri R., Hobold E., Flores L.J., Miarka B., Andrade A. (2019). Comparisons of mood states associated with outcomes achieved by female and male athletes in high-level judo and Brazilian jiu-jitsu championships: Psychological factors associated with the probability of success. J. Strength Cond. Res..

[44] Brandt R., Bevilacqua G.G., Andrade A. (2017). Perceived sleep quality; mood states; and their relationship with performance among brazilian elite athletes during a competitive period. J. Strength Cond. Res..

[45] Chapman C., Lane A.M., Brierley J.H., Terry P.C. (1997). Anxiety, self-confidence and performance in tae kwon-do. Percept. Mot. Ski..

[46] Terry P.C., Slade A. (1995). Discriminant effectiveness of psychological state measures in predicting performance outcome in karate competition. Percept. Mot. Ski..

[47] Han D.H., Kim J.H., Lee Y.S., Bae S.J., Bae S.J., Kim H.J., Sim M.Y., Sung Y.H., Lyoo I.K. (2006). Influence of temperament and anxiety on athletic performance. J. Sports Sci. Med..

[48] Parmigiani S., Dadomo H., Bartolomucci A., Brain P.F., Carbucicchio A., Costantino C., Ferrari P.F., Palanza P., Volpi R. (2009). Personality traits and endocrine response as possible asymmetry factors of agonistic outcome in karate athletes. Aggress. Behav..

[49] Cintineo H.P., Arent S.M. (2019). Anticipatory salivary cortisol and state anxiety before competition predict match outcome in Division I Collegiate Wrestlers. J. Strength Cond. Res..

[50] Arruda A.F.S., Aoki M.S., Paludo A.C., Moreira A. (2017). Salivary steroid response and competitive anxiety in elite basketball players: Effect of opponent level. Physiol. Behav..

[51] Freire G.L.M., Ferraz J.C., de Lima-Junior D., Granja C.T.L., de Oliveira D.V., do Nascimento Junior J.R.A. (2020). Anxiety in Jiu-Jitsu athletes: Differences according to age and competitive level. Res. Soc. Dev..

[52] Fernández M.M., Bello D.F., Brabec L., Brito C., Miarka B., de Durana A.L.D. (2019). State-trait anxiety and reduced emotional intelligence in combat sport athletes of different genders and competitive levels. J. Phys. Educ. Sport.

[53] Fernández M.M., Brito C.J., Miarka B., Díaz-de-Durana A.L. (2020). Anxiety and emotional intelligence: Comparisons between combat sports, gender and levels using the trait meta-mood scale and the inventory of situations and anxiety response. Front. Psychol..

[54] Selmi O., Ouergui I., Castellano J., Levitt D., Bouassida A. (2020). Effect of an intensified training period on well-being indices; recovery and psychological aspects in professional soccer players. Eur. Rev. Appl. Psychol..

[55] Shang Y., Yang S.Y. (2021). The effect of social support on athlete burnout in weightlifters: The mediation effect of mental toughness and sports motivation. Front. Psychol..

[56] Gillet N., Berjot S., Gobancé L. (2009). A motivational model of performance in the sport domain. Eur. J. Sport Sci..

[57] Jõesaar H., Hein V., Hagger M.S. (2012). Youth athletes’ perception of autonomy support from the coach, peer motivational climate and intrinsic motivation in sport. Psychol. Sport Exerc..

[58] Amorose A.J., Anderson-Butcher D., Newman T.J., Fraina M., Iachini A. (2016). High school athletes’ self-determined motivation: The independent and interactive effects of coach, father, and mother autonomy support. Psychol. Sport Exerc..

[59] Sava M.A., Panaitescu A.M. (2017). Study on the importance of attention and combativity in competitions at judoka athletes for 11–13 Years. Gymnasium.

[60] Rana M.S. (2009). Assessment of mental toughness among high and low achievers Indian wrestlers: A comparative study. Res. J. Phy. Educ. Sports Sci..

[61] Slimani M., Miarka B., Briki W., Cheour F. (2016). Comparison of mental toughness and power test performances in high-level kickboxers by competitive success. Asian J. Sports Med..

[62] Cowden R.G., Crust L., Jackman P.C., Duckett T.R. (2019). Perfectionism and motivation in sport: The mediating role of mental toughness. S. Afr. J. Sci..

[63] Skrubbeltrang L.S., Karen D., Nielsen J.C., Olesen J.S. (2018). Reproduction and opportunity: A study of dual career; aspirations and elite sports in Danish Sports Classes. Int. Rev. Sport Sociol..

