# Supplementary material for: The Role of Psychological Factors in Judo: A Systematic Review
Source: Int J Environ Res Public Health. 2022 Feb 13;19(4):2093. doi: 10.3390/ijerph19042093 (PMC8871700; doi:10.3390/ijerph19042093)
Supplement: Supplementary file 1 [file ijerph-19-02093-s001.zip › ijerph-1565960-supplementary.pdf]

Supplementary Materials: Table S1. Risk of Bias of quantitative studies through the Downs and Black checklist

	R (_/11)		EV (_/3)		IVB (_/7)		IVC (_/6)		P (_/5)		T (_/32)	
	**I1**	**I2**	**I1**	**I2**	**I1**	**I2**	**I1**	**I2**	**I1**	**I2**	**I1**	**I2**
Yoshioka, 2006	7	8	1	1	5	5	0	3	2	1	15	18
Isacco, 2019	6	7	2	1	2	5	1	2	4	1	15	17
Fortes, 2018	7	7	2	1	3	5	2	4	2	1	16	20
Koral and Dosseville, 2008	4	5	1	2	3	5	0	2	2	1	10	15
Chtourou, 2018	9	9	3	1	5	4	2	0	4	2	23	16
Gillet et al., 2010	5	6	0	3	1	4	0	0	2	4	8	17
Filaire et al., 2001	4	4	0	0	2	3	0	1	1	1	7	9
Korobeynikov et al., 2017	4	5	1	1	1	5	1	2	2	1	9	14
Hernández et al., 2009	5	5	2	1	2	5	1	2	1	1	11	14
Noce et al., 2014	6	6	0	1	2	4	0	2	2	2	10	15
Suárez-Cadenas et al., 2016	6	4	1	2	2	5	0	2	3	2	12	15
Molina et al., 2013	5	6	2	2	4	5	1	2	3	2	15	17
Kolayis et al., 2011	6	6	2	2	4	5	1	2	2	2	15	17
Filaire et al., 2001	6	6	0	1	4	3	0	0	2	1	12	11
Páez-Ardila et al., 2020	5	7	0	1	1	5	1	2	1	1	8	16
Yasar and Turgut, 2020	7	5	0	3	3	1	1	3	4	1	15	13

I1 – investigator one; I2 – investigator two; R – reporting; EV – external validity; IVB – internal validity bias; IVC – internal validity confounding; P – power; T – total score.

Table S2. Risk of Bias of quantitative studies through the JBI Appraisal Checklist

Study	Item 1	Item 2	Item 3	Item 4	Item 5	Item 6	Item 7	Item 8	Item 9	Item 10
I1										
Silva et al., 2018	Unclear	Yes	Yes	Yes	Yes	Unclear	No	Yes	Unclear	Yes
Kavoura and Ryba, 2019	Yes	Yes	Yes	Yes	Yes	Unclear	Unclear	Yes	Unclear	Yes
Gordon et al., 2021	Yes	Yes	Yes	Yes	Yes	Unclear	Unclear	Yes	Yes	Yes
I2										
Silva et al., 2018	Unclear	Yes	Yes	Yes	Yes	Unclear	No	Yes	Unclear	Yes
Kavoura and Ryba, 2019	Yes	Yes	Yes	Yes	Yes	Unclear	Unclear	Yes	No	Yes
Gordon et al., 2021	Yes	Yes	Yes	Yes	Yes	No	Unclear	Yes	Unclear	Yes

I1 – investigator one; I2 – investigator two.

Table S3. Competitive level and training experience

Study	Competitive level	Training experience
Filaire et al., 2001	Inter-regional level	10 ± 3.2 years
Filaire et al., 2001a	Inter-regional level	10 ± 3.2 years
Yoshioka et al., 2006	N.A.	N.A.
Koral and Dosseville, 2008	National and international level	9 ± 2.4 years
Hernández et al., 2009	Elite level	Average 10 years
Gillet et al et al., 2010	National level	N.A.
Kolayis et al., 2011	N.A.	8.86 ± 3.84 years
Molina et al., 2013	Elite level	N.A.
Noce et al., 2014	Elite level	N.A.
Suárez-Cadenas et al., 2016	Elite — Sub elite and veterans	10.12 ± 3.19 years
Korobeynikov et al., 2017	National level	N.A.
Chtourou et al., 2018	Elite level	N.A.
Fortes et al., 2018	National level	N.A.
Silva et al., 2018	N.A.	11.3 ± 2.2 years
Isacco et al., 2019	National level	Average 15 years
Kavoura and Ryba, 2019	Elite level	N.A.
Páez-Ardila et al., 2020	Regional level	N.A.
Yasar and Turgut, 2020	Elite level	N.A.
Gordon et al., 2021	Elite level	N.A.

N.A. – Not available
